# Memantine versus Methylphenidate in Children and Adolescents with Attention Deficit Hyperactivity Disorder: A Double-Blind, Randomized Clinical Trial

**Published:** 2015-04

**Authors:** Mohammad Reza Mohammadi, Soleiman Mohammadzadeh, Shahin Akhondzadeh

**Affiliations:** 1 Psychiatry and psychology Research Center, Roozbeh Hospital, Tehran University of Medical Sciences, Tehran, Iran; 2 Department of psychiatry, Kurdistan University of Medical Sciences

**Keywords:** *Attention Deficit Hyperactivity Disorder (ADHD)*, *Children*, *Methylphenidate (MPH)*, *Memantine*, *Clinical Trial*.

## Abstract

**Objectives:** The aim of this randomized clinical trial was to assess the efficacy of memantine versus methylphenidate in the treatment of children with attention deficit hyperactivity disorder.

**Method:** Forty participants (34 boys and 6 girls) aged 6-11 who were diagnosed with attention deficit hyperactivity disorder based on (DSM-IV-TR) criteria were selected for this study. The participants were randomly assigned to two groups: group one (n = 22) received memantine and the other group (n = 18) received methylphenidate for six weeks. Treatment outcomes were assessed using the Attention Deficit Hyperactivity Rating Scale and Clinical Global Impression- Severity Scale administered at baseline and at weeks 3 and 6 following the treatment. Also, a two-way repeated measures analysis of variance (time- treatment interaction) was used.

**Results:** At 6 weeks, methylphenidate produced a signiﬁcantly better outcome on the Parent Rating Scale scores and Clinical Global Impression- Severity than memantine. Side effects were observed more often in the memantine group. However, with respect to the frequency of side effects, the difference between the memantine and methylphenidate groups was not signiﬁcant. The most common side effects associated with memantine are appetite suppression, headache, vomiting, nausea and fatigue.

**Conclusion:** The results of this study revealed that although memantine was less effective than methylphenidate in the treatment of attention deficit hyperactivity disorder, it may be considered as an alternative treatment.

Attention deficit hyperactivity disorder (ADHD) is the most commonly diagnosed psychiatric problem in children. According to the Diagnostic and Statistical Manual of Mental Disorders—Fourth Edition (DSM-IV), this disorder is characterized by symptoms of inattention, hyperactivity and impulsivity (1).

ADHD is a heterogeneous disorder that carries a high risk of comorbidity, and it typically continues from childhood to adolescence and adulthood (2).

The neurobiology of ADHD is not completely understood, although imbalances in dopaminergic and noradrenergic systems have been implicated in the core symptoms that characterize this disorder (3).

Several studies have previously found that central nervous system stimulants, especially methylphenidate (MPH) are the primary choice of drugs in the treatment of ADHD (4-5). However, 

more recent studies have shown that long acting preparations such as the sustained-release form of dextro- amphetamines (Dexedrine Spansule) are the preferred agents (6-7). A study showed that modified- release methylphenidate, administered once daily in the morning, is effective and safe in controlling ADHD symptoms throughout the school day (8).

Children suffering from ADHD respond differentially to treatment with methylphenidate (9-10). Furthermore, the drug’s effects may only last as long as the medication is administered (11). In reality, approximately three-quarters of children respond to the first stimulant medication trial (12-13-14). Several reasons exist for the consideration of medications other than stimulant drugs in the treatment of ADHD; namely, such as stigmatization that arises from the ingestion of a controlled substance, as children treated with standard release stimulant medications should typically take at least 1 dose during school hours. Alternative medications that have been studied in the treatment of ADHD include bupropion (15), clonidine (16), guanfacine (17), theophylline (3), modafinil (18), amantadine (19), selegilin (20) and venlafaxine (21).

The consideration of these alternative medications may increase the rate of patients who respond to treatment. Clinicians and researchers are still looking for a medication that has the following characteristics: an immediate onset with benefits throughout the day, few or no side effects, lacking the potential for abuse, effective for most patients and being relatively inexpensive (22- 3).

Several lines of research suggest that the neurotransmitter glutamate may play a role in the pathophysiology of ADHD and was considered as a hypoglutamatergic condition affecting primarily prefrontostriatal pathways (23). Levels of glutamate in the prefrontal cortex and striatum of pediatric patients with ADHD are elevated and return to normal following treatment with standard ADHD therapies (stimulants or atomoxetine) (24- 25). The provided initial evidence suggests that glutamate concentrations were, in fact, raised in the left striatum of male ADHD (combined subtype) subjects at baseline compared to controls, with no increase in the prefrontal cortex (26). Elevated levels of glutamate may increase the activity of glutamate receptors, which include-amino-3-hydroxy-5-methyl-4-isoxazole-propionate (AMPA) and N-methyl-D-aspartate (NMDA) receptors. Involvement of the NMDA receptor in ADHD has been suggested by both genetic analysis and studies of signaling between the dopaminergic and glutamatergic systems (27-28). Thus, drugs that modulate AMPA and/or NMDA receptor activity may be effective treatments for ADHD.

In 2003, the FDA approved memantine for the treatment of Alzheimer's disease. This agent is the first non-cholinesterase inhibitor indicated for this disease. Unlike the cholinesterase inhibitors, which are indicated for the mild to moderate stages of Alzheimer's disease, memantine is indicated for the moderate to severe stages of the disease (29-30-31) 

Pharmacokinetic studies in adults have demonstrated that memantine is absorbed completely from the gastrointestinal tract and achieves peak Tmax within 3 to 7 hours. Protein binding is low from 42 to 45 percent and readily crosses the blood–brain barrier with a CSF serum concentration of .52. The elimination half-life of memantine is between 60 to 80 hours and is excreted unchanged in the urine. Steady-state conditions are reached in about 21 days. Memantine is largely excreted unchanged in the urine and the remaining is converted to three minimally active polar metabolites (32-33-34). In adults, the pharmacokinetic profile of memantine is not affected by food or gender, and renal clearance involves active tubular secretion moderated by pH-dependent tubular reabsorption (35).

For Alzheimer’s disease, the recommended target dose of memantine is 20 mg/day (10 mg b.i.d.), achieved from a starting dose of 5 mg/day by upward titration in weekly 5-mg increments (35). In a pooled analysis of placebo-controlled clinical trials in adult dementia populations with doses up to 20 mg/ day (10 mg b.i.d.), the safety of memantine was comparable to placebo, as indicated by similar adverse event profiles and frequency of discontinuation due to adverse events ( 29-30-31-35- 36- 37). The most common side effects with memantine were constipation, dizziness, headache and confusion. These effects were usually mild and transient and did not result in a significant dropout rate during clinical trials (38).

Robert L. and et al. in an open-label, dose-finding, 8-week trial of memantine in outpatients 6–12 years of age with ADHD combined type reported that memantine dose of 20 mg/day may be a safe and possibly effective treatment for pediatric ADHD. There were no discontinuations due to adverse events (AEs), serious AEs, deaths or suicides. Most AEs were mild and occurred during the first week of treatment. (39).

Surman CB and et al. in an open label, 12-week trial reported that memantine was largely well-tolerated and associated with improvement in ADHD symptoms and neuropsychological performance. A total of 44% of the participants had CGI ratings of much or very much improved. There were no severe adverse events, but mild adverse events were common and six participants discontinued the treatment due to adverse effects (40).

The main aim of this study was to evaluate the treatment efficacy of memantine in children and adolescents suffering from ADHD compared to treatment with methylphenidate.

## Material and Methods

*Trial Setting*

This was a 6-week, parallel group, randomized clinical trial conducted in an outpatient child and adolescent clinic at Roozbeh Psychiatric Hospital in Tehran (Iran) during March 2012 to May 2013.

*Participants*

The participants included 40 outpatients (34 boys and 6 girls) between the ages of 6 and 11 who met the DSM- IV-TR diagnostic criteria for ADHD. At screening, the researchers conducted a psychiatric evaluation with the DSM-IV-TR criteria for ADHD, and the Kiddie Schedule for Affective Disorders and Schizophrenia-Present and Lifetime diagnostic interview; then, they obtained a complete medical history and physical examination (41-42). Additional inclusion criteria included total and/or subscale scores on Attention-Deﬁcit/Hyperactivity Disorder Rating Scale IV (ADHD-RS-IV) School Version at least 1.5 standard deviations above norms for the patient’s age and gender (43). The patients were recruited from the outpatient child and adolescent clinic at Roozbeh Psychiatric Hospital. The diagnosis of ADHD was conﬁrmed by a child and adolescent psychiatrist before participants were entered into the study. All patients had combined subtype of ADHD and were newly diagnosed. Parents were carefully interviewed and asked to rate the severity of the DSM-IV-TR ADHD symptoms that their children displayed at home. Children were excluded if they had a history or current diagnosis of pervasive developmental disorders, schizophrenia or other psychiatric disorders (DSM-IV axis I), any current psychiatric comorbidity that required pharmacotherapy, or any evidence of suicide risk and mental retardation (IQ < 70). In addition, patients were excluded if they had a clinically signiﬁcant chronic medical condition, including organic brain disorder, seizures and current abuse or dependence on drugs in the last 6 months. Additional exclusion criteria were hypertension or hypotension. To participate, parents and children had to be willing to comply with all requirements of the study. After providing explanations about the procedures and purpose of the study, written informed consent was obtained from each patient’s parent or guardian. In accordance with the ethical standards of the Institutional Review Board (IRB) and also in accordance with the Helsinki declaration of 1975, revised in 2000, informed consent was received before the administrating any study procedure or supplying any medication. 

*Study Design*

Patients underwent a standard clinical assessment comprising a psychiatric evaluation, a structured diagnostic interview, a medical history and an electrocardiogram (ECG). Patients were randomized to receive tablet of memantine (Ebixa) or methylphenidate (Ritalin) in a 1:1 ratio using a computer-generated code. Both tablets were encapsulated and identical. The assignments were kept in sealed, opaque envelopes until the time of data analysis. The randomization and allocation process was done by a pharmacist at Roozbeh Hospital. All study participants were randomly assigned to receive treatment, using capsules of memantine at a dose of 10–20 mg/day (group 1) or ritalin at a dose of 20–30 mg/day depending on weight (20 mg/day for <30 kg and 30 mg/day for >30 kg (group 2) for a 6 week double blind, randomized clinical trial. Memantine was titrated up during the trial according to the following schedule: Week 1: 10 mg/day (5 mg in the morning and 5 mg at midday); week 2: 20 mg/day (10 mg in the morning and 10 mg at mid-day).

Methylphenidate was titrated up during the trial according to the following schedule: Week 1: 10 mg/day (5 mg in the morning and 5 mg at midday); week 2: 20 mg/day (10 mg in the morning and 10 mg at midday); and week 3: 30 mg/ day for children >30 kg (10 mg in the morning, 10 mg at midday and 10 mg at 16.00 h). In this schedule, drugs were blindly administered during titration. The person who administrated the medications, the rater and the patients along with their parents were blind to group assignments throughout the study. The principal measure of outcome was the Parent ADHD Rating Scale-IV (18-43-44-45-46), and the Clinical Global Impression- Severity Scale (47), both of which have been used extensively in Iran in school-age children and provide valid measures of behavioral abnormality. Attention ADHD-RS-IV is an instrument that assesses the 18 symptoms of ADHD as deﬁned in the DSM-IV- TR according to a 4-point Likert scale. The mean decrease in ADHD-RS-IV score from baseline was used as the main outcome measure of the response of ADHD treatment. The CGI-S is a 7-point scale that assesses the overall clinical status of a subject, with scores ranging from 1 (not ill) to 7 (extremely ill) (47). Patients were assessed at baseline and 21 and 42 days after the medication was started by a fellow of child psychiatry. Side effects were systematically recorded throughout the study and were assessed using a checklist that comprises 20 side effects including psychic, neurologic, autonomic and other side effects, administered by a child psychiatrist on days 7, 21 and 42. Seven patients dropped out from the Ebixa group and one dropped out from the Ritalin group (no parent collaboration or side effect), so only 32 patients completed the trial. Body weight and vital signs were measured at baseline and weeks 1, 3 and 6; and 12-lead ECG and physical examinations were evaluated at baseline and week 6.

*Statistical Analysis*

A two-way repeated measures analysis of variance (time–treatment interaction) was used. The two groups (Ebixa and methylphenidate) as a between- subjects factor (group) and the three measurements during treatment as the within-subjects factor (time) were considered. This was done for the Parent ADHD Rating Scale-IV and Clinical Global Improvement- Severity Scale scores. The results are presented as mean± SD. Differences were considered signiﬁcant with p ≤ 0.05. To compare the demographic data and frequency of side effects between the protocols, Fisher’s exact test was performed. To consider the ﬁnal differences between the two groups, at least a score of 5 on the Parent ADHD Rating Scale, S = 5 and power = 0.8, the sample size was calculated 15 patients in each group. Intention to treat analysis with the last observation carried forward procedure was performed. Response to treatment was considered as those with at least 50% decreases in Parent ADHD Rating Scale score between baseline and treatment culmination.

## Results

No signiﬁcant difference was found between patients randomly assigned to the two groups with regards to basic demographic data including age, gender and weight (Table 1).

**Figure 1:**
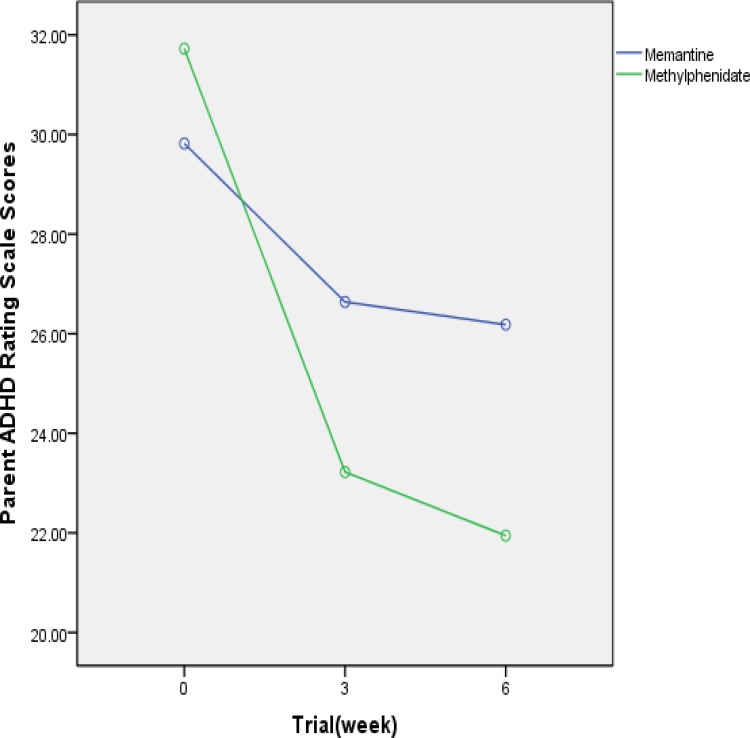
Mean SD scores of two protocols on the Parent ADHD Rating Scale-IV

**Figure 2:**
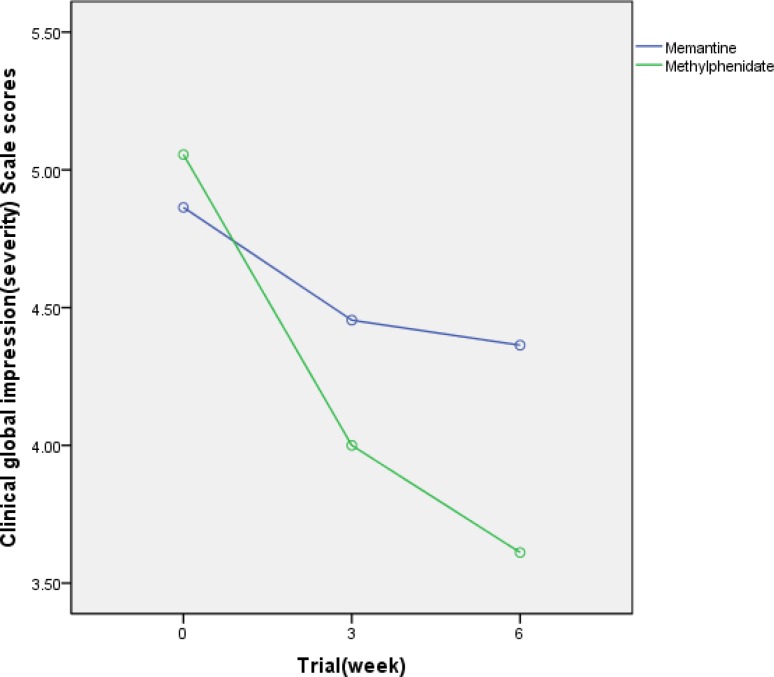
Mean SD scores of two protocols on the Parent ADHD Rating Scale-IV

**Table 1:** Comparisons of demographic and psychiatric variables in two groups of Memantine and Methylphenidat

	**Memantine group**	**Methylphenidate group**	**P value**
Girl,n	2	4	
Boy,n	20	14	
Age (mean ± SD)	9.09±1.94	8±1.32	0.051
Weight (mean ± SD)	32.4±10.46	29.33±7.65	0.3
ADHD-IV total score, mean ±SD	29.81±7.28	31.72±7.6	0.42
CGI-S rating score, mean ± SD	4.86±0.77	5.05±0.87	0.46

**Table 2:** ADHD Rating Scale-IV and Clinical global impression- severity Scale scores of study participants (inattentive and hyperactive/impulsive subscales)

	**Memantine**	**Methylphenidate**
Parent ADHD Rating Scale(inattention) Week( Baseline) Week (3) Week(6)	14.9±3.3 13.5±5.1 13.9±4.8	17.1±4.2 11±3.5 10.8±4.4
Parent ADHD Rating Scale(Hyperactivity/Impulsivity) Week( Baseline) Week (3) Week(6)	14.9±5.1 13±6.9 12.3±6.03	14.5±5.1 12.05±5.8 10.9±4.2

**Table 3:** Clinical complications and side effects in two groups of Memantine and Methylphenidat treatment

**Complication**	**methylphenidate**	**Memantine**	**P value**
abdominal pain	0	1	1.0000
appetite loss	5	6	1.0000
emotional liability	1	1	1.0000
irritability	7	3	0.1401
restlessness	4	2	0.3810
fatigue	2	3	1.0000
headache	1	3	0.6133
sadness	1	0	0.4500
Trouble in sleeping	2	1	0.5976
Tic	1	1	1.0000
vomiting	2	3	1.0000
nausea	2	3	1.0000

*Parent ADHD Rating Scale*

The mean ± SD scores of the two groups are shown in Figure 1 and Table 2 (subscales). There was no signiﬁcant difference between the two groups at day 0 (baseline) on the Parent ADHD Rating Scale (t = 0.8; df = 38; P = 0.42). The difference between the two groups was not signiﬁcant as indicated by the effect of group, the between subjects factor (df =1; F = 0.6; P = 0.443) (total score). The behaviors of the two treatment groups were not similar across time (groups by time interaction, Greenhouse–Geisser, df =1.92; F = 4.79; P = 0.01). The differences between the two protocols were not signiﬁcant at the endpoint (t =1.46; df = 38; P = 0.15) (total score). A signiﬁcant difference was observed on the reduction of scores of the Parent ADHD Rating Scale at Week 6 compared to baseline in the two groups (t = 2.8; df = 38; P = 0.01). 

Clinical Global Impression- Severity Scale

The mean ± SD scores of the two groups are shown in Figure 4 and Table 2 (subscales). There were no signiﬁcant differences between the two groups at day 0 (baseline) on the Clinical Global Impression- Severity Scale (t = 0.73; df = 38; P = 0.46). The difference between the two groups was not signiﬁcant as indicated by the effect of group, the between subjects factor (df =1; F =1.3; P = 0.25) (total score). The behaviors of the two treatment groups were not similar across time (groups by time interaction, Greenhouse–Geisser, df =1.5; F = 6.8; P = 0.04).

The differences between the two protocols were not signiﬁcant at the endpoint (t =1.9; df = 38; P = 0.053) (total score).

A signiﬁcant difference was observed on the reduction of scores of the Clinical Global Impression- Severity Scale at Week 6 compared to baseline in the two groups (t = 3.05; df = 38; P = 0.04).

*Clinical Complications and Side Effects*

A number of probably related the drugs side effects were studied (Table 3). Twelve side effects were observed over the trial, but all of them were mild to moderate and tolerable. The difference between the memantine and methylphenidate groups was not signiﬁcant in the frequency of side effects.

## Discussion

ADHD is a neurobehavioral condition with symptoms that include excessive restlessness, poor attention and impulsive acts (48-49). Most children with ADHD are referred for care because of impairment in academic, family and/or peer relationship functioning (50).

 Estimates show that 3% to 7% of school-age children and about 4% of adults have ADHD (51). Despite the well- established efﬁcacy and safety of stimulants for ADHD, alternative medications are still needed for several reasons (48-49). Almost 10% to 30% of those who are affected with ADHD may not respond to stimulants or may not be able to tolerate the associated side effects such as appetite suppression, sleep disturbance, mood difﬁculties or exacerbation of comorbid tic disorders (52). Many antidepressants affect the brain chemicals dopamine and norepinephrine that are thought to play a role in ADHD; therefore, their use in the treatment of ADHD has been studied to some degree (53- 54-55).

It has been reported that memantine is possibly effective in the treatment of ADHD (27-28-56).

Clinical characteristics of the patients, such as sex, age and weight and type of ADHD did not differ between groups and could not be considered as confounding factors. 

The results of this study suggest that administration of memantine has beneﬁcial effects for treatment of ADHD (hyperactivity type) in children and adolescents.

In this double blind, randomized, controlled study of children with ADHD, the authors detected a statistically signiﬁcant effect of memantine and methylphenidate on ADHD.

No signiﬁcant difference was observed between the two groups on the Parent Rating Scale scores and Clinical global impression- severity Scale. However, the behaviors of the two treatment groups were not similar across time, but Ritalin was more effective. The present results are in agreement with previous studies that have indicated a positive effect of memantine in the treatment of ADHD.

With respect to the side effects, no serious side effects were experienced among the memantine or methylphenidate groups. The most common side effects associated with memantine were appetite suppression, headache, vomiting, nausea and fatigue; and common side effects of methylphenidate included appetite suppression and irritability. Thus, children and adolescents suffering from ADHD may benefit from treatment with memantine as an alternative to the traditional stimulant medications employed in the treatment of ADHD children and adolescents.

Robert L. Findling, M.D et al. in an open-label, dose-finding, 8-week, trial of memantine in outpatients 6–12 years old with ADHD combined type reported that memantine dose of 20 mg/day may be a safe and effective treatment for pediatric ADHD. There were no discontinuations due to adverse events (AEs), serious AEs, deaths or suicides. Most AEs were mild and occurred during the first week of treatment (39).

Surman CB et al. In an open label, 12-week trial reported that memantine was largely well-tolerated and associated with improvement in ADHD symptoms and neuropsychological performance. A total of 44% of the participants had CGI ratings of much or very much improved. There were no severe adverse events, but mild adverse events were common and six participants discontinued the medication due to adverse effects (40).

**Limitations**

The limitations of the present study included the lack of a placebo group and the small number of participants, which should be considered when designing research in this area in the future. However, it should be mentioned that the ethics committees of many countries do not allow the use of placebo group in children with ADHD. The results of this study should be considered as “preliminary”, but they do suggest that memantine may be beneﬁcial in the treatment of ADHD (Hyperactivity type). It will be of interest to researchers to investigate the efﬁcacy of memantine on adult ADHD as well.

## Conclusion

The results of this study suggest that although memantine was less effective than methylphenidate in treating attention deficit hyperactivity disorder, it may be considered as an alternative treatment.
